# Protein Modifications and Quality Control System: Target for Alzheimer’s Disease Therapy

**DOI:** 10.3390/ijms27104266

**Published:** 2026-05-11

**Authors:** Abdullah Md. Sheikh, Shozo Yano, Shatera Tabassum, Jubo Bhuiya, Atsushi Nagai

**Affiliations:** 1 Department of Laboratory Medicine, Faculty of Medicine, Shimane University, 89-1 Enya Cho, Izumo 693-8501, Japan; syano@med.shimane-u.ac.jp; 2 The Center for Integrated Kidney Research and Advance (IKRA), Faculty of Medicine, Shimane University, 89-1 Enya Cho, Izumo 693-8501, Japan; 3 Department of Neurology, Faculty of Medicine, Shimane University, 89-1 Enya Cho, Izumo 693-8501, Japan; tabassum@med.shimane-u.ac.jp (S.T.); m239427@med.shimane-u.ac.jp (J.B.); anagai@med.shimane-u.ac.jp (A.N.)

**Keywords:** Alzheimer’s disease, amyloid β peptide, tau, protein quality control system, posttranslational modification, neuroinflammations, neurodegeneration

## Abstract

Alzheimer’s disease (AD) is a progressive neurodegenerative disorder characterized by memory loss and cognitive decline. Its main pathological features are extracellular plaques composed of aggregated amyloid-β (Aβ) peptides and intracellular neurofibrillary tangles formed by hyperphosphorylated tau. The Aβ hypothesis proposes that Aβ accumulation is a key driver of AD, influencing tau pathology, neuroinflammation, and neurodegeneration. However, therapies that reduce Aβ have shown limited clinical benefits. This suggests that the mechanisms underlying peptide-mediated modulation of AD pathology are much more complex. Both Aβ and tau undergo various post-translational modifications (PTMs) that affect their structure, aggregation, and toxicity. In addition, these abnormal proteins are not efficiently cleared in AD, indicating dysfunction of the protein quality control (PQC) system that maintains proteostasis. Such abnormal PTMs and impaired PQC likely work together to drive disease progression, which may explain the limited success of Aβ-reduction therapies. In this review, we describe how major PTMs, including phosphorylation, ubiquitination, acetylation, glycosylation, and oxidation, regulate the pathological behavior of Aβ and tau. We also discuss the role of the PQC systems in the pathology of AD. We propose that dysregulation of PTMs and PQC constitutes a convergent mechanism underlying AD pathogenesis. Therapeutic strategies targeting these processes may provide more effective and sustained disease modification than approaches focused solely on Aβ reduction.

## 1. Introduction

Alzheimer’s disease (AD) is a common neurodegenerative disorder manifested as progressive dementia in the elderly population [1]. In its earliest stages, the disease typically manifests as a subtle yet persistent inability to form or retrieve newly acquired memories [2]. As the neurodegenerative process advances, the clinical picture broadens to encompass a diverse array of cognitive and behavioral impairments [3]. These include the attrition of episodic memory, significant language deficits, visuospatial disorientation, and a failure in executive functions such as decision-making, learning, and judgment [4]. Such a disease progression course not only decreases the self-sufficient functional ability of the affected individuals, but also poses an immense emotional, physical, and economic burden on their families, and the society at large [1].

Epidemiologically, AD affects millions worldwide [1]. The disease risk is increased with aging and certain genetic factors [2]. Over 55 million people worldwide lived with dementia in 2020, with AD being the most common form [3]. This number is expected to nearly double every 20 years, and the number is estimated to reach 139 million by 2050 [5]. In the USA, it is estimated that around 10% of people over 65 live with AD, which is similar to the global average [6]. Japan presents a unique case study in this demographic shift [1]. While the prevalence rates in Japan were once lower than those in the West, the country’s status as one of the world’s fastest-aging societies has led to a dramatic upward trend in the occurrence of the disease [2]. Previous estimates suggest a prevalence range of 2.4% to 11.0% for dementia in those over 65, but national health projections indicate that this figure could exceed 20% by 2030 [3]. Importantly, this increasing trend of dementia diseases is no longer confined to high-income countries [5]. As healthcare infrastructure improves in developing regions and life expectancy rises, the prevalence of AD is increasing in these areas as well, posing a huge and unprecedented burden on healthcare systems [6].

Despite decades of intensive research, therapeutic strategies for AD have remained largely focused on symptomatic management rather than fundamental disease modification [7]. For many years, the standard of care was limited to cholinesterase inhibitors and NMDA receptor antagonists [8,9]. While these medications provide modest, temporary improvements in cognitive function and quality of life, they do not alter the underlying neurodegenerative pathology [8,10]. Recently, antibody-mediated treatments have been introduced in the field as disease-modifying therapies [11,12]. The FDA approval of such therapies, such as lecanemab and donanemab, has generated significant hope in the clinical management of AD [8,10,11,13,14]. These monoclonal antibodies are specifically designed to target and reduce brain levels of amyloid-β (Aβ), the peptide widely considered a primary driver of AD pathogenesis [15]. While these immunotherapies show promise, particularly for patients in the early or prodromal stages, their clinical effects are generally described as moderate and shown to slow the rate of cognitive decline rather than halting or reversing the disease entirely [15,16]. This limitation suggests that while Aβ is a critical factor, its removal alone might not be sufficient for disease management [3,17]. Consequently, there is a pressing need for continued research to identify novel therapeutic targets rooted in the broader molecular pathology of AD [7]. Emerging studies demonstrate that the Aβ peptide undergoes several post-translational modifications and structural transitions [8,18]. Also, failure of the protein quality control system (PQC) is well-documented in AD brains [18,19]. These changes might be pivotal in the development and maturation of AD pathology [10,18,19]. In this review, we aim to discuss the PQC and various post-translational modifications of Aβ, evaluate their relevance to AD pathology, and outline future research directions towards the development of more comprehensive and effective disease-modifying therapy [4,5,12,14,17,18,19,20].

## 2. Physiological Role of Aβ

Aβ peptides are generated from the amyloid precursor protein (APP) through a process of sequential proteolytic cleavage [21]. This process begins with β-secretase (BACE1), which cleaves APP to release the soluble sAPPβ fragment and a membrane-bound C-terminal fragment (CTF-β) [21,22]. Subsequently, the γ-secretase complex cleaves this remaining fragment to liberate various Aβ species, including Aβ_1–40_ and Aβ_1–42_ [21,22,23]. Among these, Aβ_1–42_ exhibits the strongest propensity for aggregation [21,22,23,24].

Traditionally, Aβ has been regarded as a byproduct of the enzymatic processing of APP, with no clear physiological function [25,26]. However, the evolutionary conservation of both APP and Aβ sequences suggests that this peptide serves an important biological role [27]. In support of this concept, accumulating evidence indicates that Aβ possesses antimicrobial activity [28,29]. Aβ deposits are observed in cognitively normal individuals, where it is colocalized with bacterial components like LPS [27,30]. These findings support the idea that neuron-derived Aβ may function as an innate immune effector that aggregates in response to invading pathogens [31,32].

Several mechanisms have been proposed to explain the antimicrobial properties of Aβ [25]. Firstly, Aβ peptides may exert direct microbicidal effects by inserting into microbial membranes and forming pore-like structures, leading to membrane disruption and cell death [25,26]. Secondly, Aβ promotes pathogen agglutination [27]. The intrinsic propensity of Aβ to form oligomers and fibrils enables the peptide to bind to microbial surface components, including LPS or lipoteichoic acid, thereby trapping pathogens and limiting their spread [28]. In this role, Aβ acts as an antimicrobial peptide (AMP), with its aggregation into amyloid fibrils immobilizing pathogens in a manner similar to human antimicrobial peptides such as LL-37 or structures like neutrophil extracellular traps (NETs) [33]. In addition, Aβ may act as an opsonin and enhance the recognition and phagocytosis of microbes by innate immune cells, including microglia [29]. Aβ has also been implicated in modulating immune responses by stimulating the production of inflammatory mediators and recruiting immune cells to sites of infection [34]. Collectively, these observations indicate that Aβ is an active component of the innate immune defense system of the brain [25].

## 3. AD Pathology

The pathological features of AD can be defined as a complex interplay of protein misfolding, impaired clearance, and chronic inflammation [35,36]. The definitive markers for a post-mortem AD diagnosis include the presence of extracellular amyloid plaques, intracellular neurofibrillary tangles (NFTs), area-specific and neuron-type-specific degeneration, and persistent neuroinflammation [37,38,39].

### 3.1. The Aβ Cascade

Amyloid plaques primarily contain Aβ peptides [35,36,37]. In addition to Aβ, these plaques may contain metals, inflammatory cells, and fragments of dead nerve cells [21,38,39]. Importantly, amyloid plaques also contain other proteins, including apolipoprotein E (ApoE) [22,23,24]. Around the plaque, inflammatory glial cells, especially activated microglia, are seen [40,41]. Astrocytes, microglia, and other central nervous system cell types act in a highly coordinated manner in the pathogenesis of AD. In the healthy brain, astrocytes support synaptic function through glutamate clearance and metabolic coupling with neurons, while microglia survey the microenvironment and maintain tissue homeostasis [40,41,42]. In AD, Aβ accumulation and tau pathology disrupt this balance, triggering reactive astrogliosis and sustained microglial activation. These changes promote the release of pro-inflammatory cytokines and chemokines, which not only impair Aβ clearance but also exacerbate neuronal stress and synaptic dysfunction [21,38,39,42]. Neurons, in turn, accumulate toxic Aβ and hyperphosphorylated tau, leading to progressive synaptic loss and cell death [31,42,43,44]. Concurrently, oligodendrocyte dysfunction and myelin abnormalities further compromise axonal integrity [45]. Thus, dynamic crosstalk among astrocytes, microglia, neurons, and oligodendrocytes establishes a self-amplifying cycle of neuroinflammation, proteostatic failure, and neurodegeneration in AD [40,41,45,46].

Within plaques, Aβ adopts fibrillar structures characterized by antiparallel β-sheet conformations. During the aggregation process, the peptide transitions through several structural states, from soluble monomers to highly toxic oligomers and small protofibrils, eventually forming mature β-sheet fibrils [40]. Although fibrillar aggregates are a defining feature of AD, accumulating evidence indicates that the intermediate oligomeric species are the most neurotoxic [12,41]. Given that aggregated Aβ exhibits antimicrobial activity, the peptide aggregation may initially represent a protective response to microbial invasion rather than a purely AD pathological process [26]. A pathology may emerge when this innate immune mechanism becomes dysregulated [28]. One potential contributor to such dysregulation could be post-translational modifications [29]. Notably, various posttranslational systems are frequently changed under chronic inflammatory conditions [34]. These modifications may impair the clearance of Aβ-pathogen complexes [46]. Also, the immune regulatory properties of the complex can be changed, resulting in the persistence and sustained activation of neuroinflammatory pathways [30]. In this context, AD can be viewed as the consequence of a normally protective innate immune response that becomes maladaptive due to persistent dysregulation and impaired resolution, potentially driven in part by PTMs of Aβ [31]. This perspective provides important insights into disease mechanisms and may be important for the development of new therapeutic strategies [32].

### 3.2. Dynamics of Aβ: Production vs. Clearance

The accumulation of Aβ in the brain is essentially a failure of homeostasis, a disruption in the balance between the production and clearance of the peptide [44]. In familial AD, this balance is typically influenced by rare genetic mutations in APP or the presenilin genes (components of γ-secretase) that lead to the overproduction of Aβ [47]. Numerous animal and genetic studies have underscored the significance of these pathways in disease pathogenesis [48]. For instance, longitudinal animal models have demonstrated that the overproduction of Aβ through gene manipulation leads to a predictable cascade of neuroinflammation, neurodegeneration, and the cognitive impairments characteristic of human AD [49]. While these increased-production mutations define familial AD, the much more common sporadic form of the disease lacks this specific genetic explanation [50]. In these patients, the pathology is instead driven by compromised clearance of the peptide [51]. Aβ is removed from the brain in several ways. After production, the Aβ peptide can be metabolized by several enzymes, including neprilysin, insulin-degrading enzyme, matrix metalloproteases, and angiotensin converting enzyme 2 (ACE 2). ACE 2 is particularly important because it has been shown that its activity is significantly reduced in the AD brain [52]. ACE 2 metabolizes toxic Aβ_1–43_ to less toxic forms like Aβ_1–40_ [53]. Also, through the production of angiotensin-(1-7), it shows neuroprotective, anti-inflammatory, and anti-fibrotic effects, and reduces Aβ-induced inflammatory damage [54]. Aβ is also removed from the central nervous system via the perivascular (glymphatic) system [44]. When this drainage is hindered, the peptide reaches a critical concentration and begins to aggregate [47]. ApoE plays a pivotal role in this clearance mechanism [48]. Genome-wide association studies (GWAS) have consistently identified the *ApoE4* variant as the most significant genetic risk factor for sporadic AD [49]. Research indicates that the ApoE4 isoform binds to Aβ in a way that is cleared far less efficiently through the blood–brain barrier than the ApoE2 or ApoE3 variants [50]. Consequently, individuals carrying the ApoE4 allele experience an earlier and more aggressive accumulation of amyloid plaques, creating a toxic environment that ultimately accelerates synaptic loss and neuronal death [51].

### 3.3. Tau Pathology and Cytoskeletal Changes

While Aβ often initiates the pathological cascade, the formation of neurofibrillary tangles (NFTs) is equally critical to the progression of dementia [55]. These tangles are composed of hyperphosphorylated tau, a microtubule-associated protein predominantly expressed in neurons [56]. In a healthy brain, microtubules serve as the primary structural framework and act as a physical pathway for transporting organelles, vesicles, and proteins across the long distances of the axon [57]. Under physiological conditions, tau undergoes a tightly regulated cycle of phosphorylation and dephosphorylation, the posttranslational modifications that ensure its proper binding and stabilization of these microtubules [58]. However, in an AD brain, the delicate balance between specific kinases (such as GSK-3β, CDK5, and PKA) and phosphatases (primarily PP2A) is disrupted [59]. This enzymatic dysregulation leads to the abnormal hyperphosphorylation of tau, causing it to lose its affinity for microtubules [60]. Consequently, tau detaches and aggregates into paired helical filaments (PHFs), which eventually coalesce into the dense, insoluble NFTs [61]. The consequences of this detachment are profound [62]. The loss of functional tau results in the destabilization of the neuronal cytoskeleton and directly impairs synaptic communication and axonal transport [63]. Furthermore, hyperphosphorylated tau has been shown to interact directly with mitochondrial proteins, impairing energy production and triggering an increase in oxidative stress [55]. Ultimately, this combination of dysfunctional energy metabolism, widespread synaptic loss, and structural cellular failure serves as the final common pathway toward the progressive cognitive decline observed in patients [56]. As these tangles spread through the cortex in a predictable pattern, they correlate more closely with the severity of clinical symptoms than amyloid plaques alone, marking the transition from cellular dysfunction to irreversible neurodegeneration and dementia [57].

### 3.4. Mechanisms of Neurodegeneration and Neuroinflammation

#### 3.4.1. Glial Activation and the Inflammatory Cycle

Neuroinflammation is a primary mediator of Aβ and tau-induced damage [51]. Initially, microglia serve a neuroprotective role by phagocytosing aggregates [64]. However, chronic exposure to oligomeric Aβ and extracellular tau fragments, acting as damage-associated molecular patterns (DAMPs), shifts microglia toward a pro-inflammatory M1 phenotype [65]. These cells release potent cytokines (TNF-α, IL-1β, IL-6) and ROS, creating a self-reinforcing cycle of inflammation that further promotes neurodegeneration [66].

#### 3.4.2. Synaptic Dysfunction and Excitotoxicity

Synaptic loss is the most direct correlate of cognitive impairment in AD [67]. Elevated Aβ levels trigger the internalization of N-methyl-D-aspartate (NMDA) receptors via the activation of the tyrosine phosphatase STEP [68]. Furthermore, Aβ oligomers can insert into neuronal membranes to form calcium-permeable pores, leading to chronic calcium influx [69,70]. This calcium dysregulation, coupled with Aβ-mediated degradation of receptors like EphB2, severely impairs long-term potentiation (LTP) and promotes dendritic spine loss in AD [71].

#### 3.4.3. Mitochondrial Failure and Oxidative Stress

Aβ and tau aggregates directly impair mitochondrial function by blocking the transport of nuclear-encoded proteins and disrupting the electron transport chain [72,73,74]. Furthermore, redox-active metal ions (copper and iron) bound to Aβ can catalyze the Fenton reaction, producing hydroxyl radicals [74,75]. The resulting oxidative stress damages neuronal lipids and DNA, while simultaneously exhausting antioxidant defenses like glutathione (GSH), ultimately culminating in apoptotic neuronal death [75,76,77].

## 4. Post-Translational Modifications of Proteins

Post-translational modifications (PTMs) represent a sophisticated method of biological regulation that occurs after a protein has undergone ribosomal synthesis and folding [18,55]. Through covalently attaching functional groups, proteins, or complex sugars to specific amino acid side chains, the hydrophobicity, tertiary structure, enzymatic activity, and half-life of a protein can be dramatically altered [18,31,58]. This process effectively expands the functional diversity of the proteome far beyond what is encoded by the genome alone [18,59]. While the human genome contains approximately 30,000 genes, the functional proteome is estimated to be several orders of magnitude larger due to the vast array of possible PTM combinations [60,78]. These modifications are not merely static markers; they are dynamic, often reversible, and highly sensitive to the cellular microenvironment [61,79]. Major PTMs include phosphorylation, acetylation, ubiquitination, glycosylation, methylation, and oxidation [62,78]. Each of these plays a specialized role [49]. For example, phosphorylation is primarily involved in intracellular signaling, acetylation acts as a master regulator of the epigenetic landscape, and ubiquitination serves as the primary gatekeeper for protein degradation and quality control [58]. The biological significance of PTMs is underscored by their involvement in nearly every major cellular transition, from cell cycle checkpoints to the initiation of immune responses [18,59]. Consequently, when the enzymes responsible for adding or removing these modifications become dysregulated, the results are often catastrophic [60]. Aberrant PTMs are hallmark features of diverse human pathologies, including various cancers, Type 2 Diabetes, and neurodegenerative disorders [61,80]. In AD, for instance, the pathological hyperphosphorylation of the microtubule-associated protein tau leads directly to the formation of neurofibrillary tangles, a hallmark pathology of the disease [62]. Understanding the molecular basis of these modifications is therefore essential for the design of next-generation targeted therapies [18,55,79,80].

### 4.1. Phosphorylation

Protein phosphorylation is perhaps the most extensively studied PTM, acting as a universal molecular switch that regulates roughly one-third of all proteins in a eukaryotic cell [18,31,44,77,80]. This reversible process involves the transfer of a γ-phosphate group from Adenosine Triphosphate (ATP) to the hydroxyl group of specific residues, most commonly serine, threonine, or tyrosine [31,43,81]. This reaction is catalyzed by a vast family of enzymes known as protein kinases. Conversely, the removal of these phosphate groups is mediated by protein phosphatases [58,59,81]. In physiological terms, the addition of a phosphate group introduces a localized negative charge, which can induce conformational changes in the protein, thereby masking or unmasking functional domains [43,81]. For example, when a growth factor binds to its cognate receptor on the cell surface, it triggers a phosphorylation cascade where kinases sequentially activate one another, eventually leading to the translocation of transcription factors into the nucleus to initiate cell division [18,43,79]. Beyond signaling, phosphorylation is a critical regulator of metabolic flux. In the liver, the enzyme glycogen phosphorylase, responsible for breaking down glycogen into glucose, is activated by phosphorylation in response to glucagon or adrenaline [18,43,80,82]. This allows the body to rapidly mobilize energy reserves during periods of fasting or stress. In the context of the nervous system, phosphorylation at the synapse modulates the conductance of ion channels and the docking of neurotransmitter vesicles, processes that are fundamental to synaptic plasticity and the formation of long-term memories [83]. When this phosphorylation system is dysregulated, the cell tries to adapt [43,58,83]. However, persistent dysregulation and failure of adaptation cause the development of diseases. In many forms of cancer, mutations in tyrosine kinases like the Epidermal Growth Factor Receptor (EGFR) lead to constitutive phosphorylation, sending a continuous, erroneous signal to the cell to divide [43,58,82,83]. Similarly, in AD, the overactivity of kinases such as GSK-3β results in the hyperphosphorylation of tau, which causes the protein to detach from microtubules, leading to cytoskeletal collapse [43,61,81,84].

### 4.2. Ubiquitination

Protein ubiquitination is another post-translational modification that regulates numerous cellular processes by tagging proteins for degradation, altering their cellular localization, or modulating their activity [85,86]. Ubiquitination involves the covalent attachment of ubiquitin, a small 76-amino acid protein, to the ϵ-amino group of lysine residues on target proteins [72,86]. This process is executed through a highly coordinated three-step enzymatic cascade [73,86]. First, the E1 ubiquitin-activating enzyme uses ATP to activate the ubiquitin molecule [20,74,86]. Second, the E2 ubiquitin-conjugating enzyme carries the activated ubiquitin. Finally, an E3 ubiquitin ligase identifies a specific substrate and facilitates the transfer of ubiquitin onto it [20,74,86,87]. The primary function of ubiquitination is to serve as a tag for a protein that is destined to the 26S proteasome, a large protease complex that shreds proteins into short peptides [73,86,88]. This ubiquitin–proteasome system (UPS) is one of the primary protein quality control systems against misfolded, damaged, or redundant proteins [85,86]. By maintaining proteostasis, UPS prevents the toxic accumulation of protein aggregates that would otherwise disrupt cellular functions [72,86,89]. However, the functions of ubiquitination are not limited to protein degradation only [73,86]. The way ubiquitin molecules are linked to one another (polyubiquitination chains) determines the fate of the protein [20,74,86]. While K48-linked chains typically target proteins for the proteasome, K63-linked chains are often involved in non-degradative signaling, such as DNA damage repair and endocytic trafficking [90,91]. For instance, the ubiquitination of surface receptors can trigger their internalization, which prevents overstimulation of the cell by extracellular ligands [90,92]. In pathological states, particularly in oncology, the UPS is frequently dysregulated [20,74,86,87]. Many tumor cells overexpress specific E3 ligases, such as MDM2, which target the tumor suppressor p53 for premature degradation [85,86,93]. This effectively hampers the ability of a cell to undergo apoptosis in response to DNA damage, allowing the cancer to proliferate unchecked [72,89].

### 4.3. Acetylation

Protein acetylation involves the transfer of an acetyl group from Acetyl-CoA to either the N-terminus of a protein or the ϵ-amino group of internal lysine residues [76]. This process is balanced by Lysine Acetyltransferases (KATs) and Lysine Deacetylases (KDACs), more commonly referred to in the context of DNA as Histone Deacetylases (HDACs) [94]. Acetylation is most famous for its role in epigenetic regulation [94,95,96,97]. In the nucleus, DNA is tightly wrapped around histone proteins to form chromatin [96]. The positive charge of histones creates a strong electrostatic attraction to the negatively charged DNA backbone [98]. When histones are acetylated, their positive charge is neutralized, causing the chromatin to relax into an open conformation known as euchromatin [82]. This provides the transcriptional machinery, such as RNA polymerase, access to the genes, thereby promoting expression [18,82]. Conversely, deacetylation leads to chromatin condensation (heterochromatin) and gene silencing [99]. Beyond the nucleus, acetylation is a major regulator of cellular metabolism and the cytoskeleton [99]. Many mitochondrial enzymes involved in the TCA cycle and fatty acid oxidation are regulated by acetylation levels, which are in turn sensitive to the availability of Acetyl-CoA, linking the metabolic state of the cell directly to its protein function [100,101]. Furthermore, the acetylation of α-tubulin is a marker of long-lived, stable microtubules [82,96,98]. Dysregulation of acetylation is a key feature of many cancers; for example, the overexpression of HDACs is often used by tumor cells to silence tumor suppressor genes, leading to the clinical development of HDAC inhibitors as a potent class of chemotherapy [9,79,94,96,100].

### 4.4. Glycosylation

Glycosylation is perhaps the most structurally complex PTM, involving the enzymatic attachment of carbohydrate chains, or glycans, to proteins [89,102]. This occurs primarily within the lumen of the Endoplasmic Reticulum (ER) and the Golgi apparatus [79,87,91]. The two most common types are N-linked glycosylation (attached to asparagine) and O-linked glycosylation (attached to serine or threonine) [103]. Physiologically, glycosylation is essential for protein folding and quality control [103,104]. Within the ER, the calnexin/calreticulin cycle uses glycan markers to determine if a nascent protein is correctly folded [103]. If a protein fails this check, its glycans are modified to signal for ER-Associated Degradation (ERAD) [89,102]. Beyond folding, glycosylation is critical for cellular identity [103]. The glycans on the surface of immune cells and red blood cells (determining ABO blood types) allow the immune system to distinguish between self and foreign proteins [89,102]. In disease, glycosylation patterns are often profoundly altered [89,102]. Cancer cells frequently exhibit hypersialylation, an overabundance of sialic acid on their surface glycoproteins [79,87,91]. This creates a negative charge that repels immune cells and masks the tumor from detection, which facilitates metastasis [103]. Furthermore, in many congenital disorders of glycosylation (CDGs), mutations in glycosyltransferases lead to severe multisystem failures, illustrating the fundamental nature of this modification [103,104].

### 4.5. Oxidation

Protein oxidation occurs when Reactive Oxygen Species (ROS), such as superoxide or hydrogen peroxide, react with amino acid side chains [75,85,105]. Traditionally viewed only as a byproduct of oxidative stress and aging, mild and reversible oxidation is now recognized as an important signaling mechanism [75,106]. For example, the reversible oxidation of cysteine residues into disulfide bonds or sulfenic acids can act as a redox-sensitive switch that alters the activity of transcription factors and phosphatases in response to the cell’s metabolic state [75,85,107]. However, when ROS production overwhelms the antioxidant defense system, irreversible oxidation, such as protein carbonylation, may occur [75,85,107]. These oxidized proteins lose their tertiary structure and often form cross-linked aggregates that are resistant to proteolysis [108,109]. This accumulation of molecular junk is one of the main features of the free radical theory of aging and is heavily implicated in the progression of neurodegeneration, where the high oxygen consumption of the brain makes it particularly vulnerable to oxidative damage [75,106,109].

### 4.6. The PTM Crosstalk

The most recent frontier in proteomics is the study of PTM crosstalk, the phenomenon where one modification influences the occurrence or function of another [48,55,75,80]. This can happen through competitive crosstalk where two different PTMs (such as acetylation and ubiquitination) compete for the same lysine residue [18,31,57,79,80]. If the site is acetylated, the protein is stabilized; if it is ubiquitinated, it is destroyed [59,60,61]. Alternatively, sequential crosstalk occurs when one PTM is required as a priming event for another [62,63]. For example, the phosphorylation of a protein often creates a degron motif that is then recognized by an E3 ubiquitin ligase for subsequent degradation [57,59,79,91]. This intricate network of modifications allows the cell to integrate multiple environmental signals into a single, refined proteomic response [78,79,80]. As we move toward a more comprehensive understanding of these systems, the PTM-ome is emerging as a primary target for therapeutic intervention. By developing drugs that can specifically modulate these PTM enzymes, we may eventually be able to reprogram diseased cells back to a state of healthy homeostasis.

## 5. The Protein Quality Control System

Most of the work a cell performs depends on proteins [76]. For specific tasks, the 3D conformation of proteins is crucial [18,94]. This is where the intricate protein quality control system (PQC) comes in [93]. PQC is a complex network of cellular pathways that meticulously regulate protein fate [61], which primarily occurs in the endoplasmic reticulum (ER), a cellular compartment where proteins are synthesized and folded [93,110,111]. For protein synthesis, ribosomes translate messenger RNA (mRNA) instructions into a chain of amino acids, forming an unfolded polypeptide [111,112]. Then the unfolded protein enters the ER, where molecular chaperones assist it in folding into the correct three-dimensional structure [20,110,112]. For quality check, the ER quality control system meticulously inspects the folded protein [93,111]. If correctly folded, the protein is transported out of the ER for its designated function [39,113]. If the proteins are misfolded or denatured in the cytoplasm afterwards, chaperones attempt to refold the protein [110]. If unsuccessful, the protein is tagged by ubiquitin and degraded by the ubiquitin–proteasome system (UPS) or targeted for autophagic breakdown [76]. Additionally, old proteins or those no longer required under altered cellular conditions are targeted for degradation [18,94]. When proteins begin to aggregate and can no longer be handled by the UPS, the autophagy–lysosomal pathway activates to eliminate the bulk of aggregated proteins and damaged organelles [106]. The components of the PQC system are discussed below.

### 5.1. Molecular Chaperones

Molecular chaperones are proteins that interact with, stabilize, and help a non-native protein to acquire its native conformation [72,114]. To achieve the native conformation of a protein, chaperones are involved in de novo folding [73,114]. Importantly, they assist in the refolding of stress-denatured proteins and are involved in oligomeric assembly [19,74]. Additionally, chaperones are shown to be involved in intracellular protein transport and assist in proteolytic degradation [75]. By performing these functions, molecular chaperones play a crucial role in maintaining cellular proteostasis, which is the balance of protein synthesis, folding, trafficking, and degradation within a cell [104,110]. Since proteins are involved in almost every cellular function, disruptions in proteostasis are implicated in various diseases, including neurodegenerative disorders such as Alzheimer’s disease [19,77,114].

The chaperone network can be broadly classified into several families, including heat shock proteins (HSPs), chaperonins, and small heat shock proteins (sHSPs) [19,114,115,116,117]. These families are characterized by their ability to bind and stabilize unfolded or misfolded protein substrates, preventing aggregation and facilitating proper folding.

(a) Heat Shock Proteins (HSPs): HSPs are a well-characterized family of molecular chaperones that are upregulated in response to cellular stress [115,116,117,118,119]. They are categorized based on their molecular weight: Hsp70, Hsp90, Hsp60, and small HSPs [19,114,115,116,117].

- Hsp70: This chaperone binds to nascent polypeptide chains and partially folded intermediates, preventing aggregation [115,120]. It collaborates with co-chaperones such as Hsp40 and nucleotide exchange factors to facilitate the correct folding of proteins [115].
- Hsp90: Hsp90 is involved in the maturation of a wide array of substrate proteins, including steroid hormone receptors and kinases [114,115]. It operates in conjunction with co-chaperones like p23, Hop, and Aha1, which regulate its ATPase activity and target protein interactions [115,121].

(b) Hsp60 (Chaperonins): Hsp60 proteins, also known as chaperonins, are involved in folding newly synthesized proteins in the mitochondria [115]. GroEL and GroES in prokaryotes and their eukaryotic counterparts, TRiC/CCT, are key representatives of this group [19,110,115,120].

(c) Small HSPs: These chaperones, such as Hsp27, form oligomeric complexes that bind unfolded proteins and prevent their aggregation, particularly under stress conditions like heat shock [19,110,120].

The prevention of protein misfolding and aggregation by molecular chaperones is achieved through several mechanisms [118]. Firstly, chaperones can bind to hydrophobic regions of unfolded or partially folded proteins [115,120]. Since hydrophobic interaction is one of the main mechanisms of protein aggregation, binding to the hydrophobic regions shields the proteins from such changes [115]. They also facilitate the correct folding of proteins through their ATPase activities, which prevents aggregation and promotes proper interactions [115,121]. Some chaperones can recognize misfolded proteins and refold them [118]. When proteins cannot be refolded, chaperones can direct them to degradation pathways, such as the ubiquitin–proteasome system (UPS) or autophagy [88,118,119].

### 5.2. The Ubiquitin–Proteasome System (UPS)

The ubiquitin–proteasome system (UPS) is a highly regulated mechanism responsible for degrading and recycling damaged or misfolded proteins within the cell [89,91]. This system involves tagging unwanted proteins with ubiquitin, a small regulatory protein, through a process called ubiquitination [92,93]. Once properly tagged, these proteins are recognized and directed to the proteasome, a large protease complex that degrades and breaks down the tagged proteins into small peptides [88,90,114]. The UPS plays a crucial role in maintaining cellular homeostasis by controlling the quality and quantity of proteins, regulating various cellular processes such as cell cycle progression, signal transduction, and stress responses [89,91,115,121].

The process of ubiquitination involves three main enzymatic steps: activation, conjugation, and ligation [89,91]. First, an E1 ubiquitin-activating enzyme activates ubiquitin in an ATP-dependent manner, forming a high-energy thioester bond between the enzyme and the ubiquitin molecule [89,91]. Next, the activated ubiquitin is transferred to an E2 ubiquitin-conjugating enzyme [89,91]. Finally, an E3 ubiquitin ligase facilitates the transfer of ubiquitin from the E2 enzyme to the target protein, often attaching ubiquitin to a lysine residue on the substrate protein [92,93]. This can result in the formation of a polyubiquitin chain, where additional ubiquitin molecules are attached to the initial ubiquitin [89,91]. The number and arrangement of ubiquitin molecules on the target protein determine its fate [89,91]. For instance, a single ubiquitin might signal a change in protein location within the cell, while a chain of ubiquitin molecules often targets the protein for degradation by the proteasome [89,91]. The specificity and regulation of ubiquitination are largely determined by the E3 ligases, which recognize specific substrate proteins, thus ensuring targeted and precise degradation within the cell [92,93].

The proteasome is a large, multi-subunit protease complex responsible for degrading ubiquitinated proteins [89,91]. Structurally, the proteasome is composed of a 20S core particle (CP) and one or two 19S regulatory particles (RP) [91]. The 20S core is a barrel-shaped structure made up of four stacked rings: two outer rings composed of seven α subunits and two inner rings composed of seven β subunits [92]. The catalytic sites are located within the inner β rings, where proteolysis occurs [89,92,93]. The 19S RP caps the ends of the 20S core and is involved in recognizing ubiquitinated substrates, unfolding them, and translocating them into the core for degradation [89]. The 19S RP consists of multiple subunits that perform distinct functions, such as binding ubiquitin chains, deubiquitinating substrates, and using ATPase activity to unfold target proteins [91]. By removing unwanted proteins, the proteasome ensures proper cellular function and prevents the accumulation of potentially toxic protein aggregates, which is vital for preventing diseases such as cancer and neurodegenerative disorders, including AD [92].

### 5.3. Autophagy–Lysosome Pathway

Autophagy is a cellular degradation process that removes damaged organelles, misfolded proteins, and other cellular debris, maintaining cellular homeostasis and responding to stress [105,106,107]. There are three primary types of autophagy: macroautophagy, microautophagy, and chaperone-mediated autophagy (CMA) [106]. In macroautophagy, cytoplasmic components are sequestered into double-membrane vesicles called autophagosomes, which then fuse with lysosomes to form autophago-lysosomes, where the contents are degraded and recycled [107,122]. This process is tightly regulated by autophagy-related (ATG) proteins [123]. Microautophagy involves the direct invagination of the lysosomal membrane to engulf cytoplasmic materials, leading to their degradation within the lysosome [122]. This process is generally less specific than macroautophagy [124]. Chaperone-mediated autophagy (CMA) is a highly selective process where specific cytosolic proteins containing a KFERQ-like motif are recognized by the chaperone protein Hsc70 [125]. These targeted proteins are then translocated across the lysosomal membrane with the help of the lysosome-associated membrane protein type 2A (LAMP-2A) [105]. Once inside the lysosome, the substrates are degraded by lysosomal proteases [106]. Each of these autophagic pathways plays a critical role in cellular quality control, adaptation to nutrient stress, and protection against various diseases, including neurodegenerative disorders and cancer [107,122].

## 6. Posttranslational Modifications, PQC, and Their Implications in Alzheimer’s Disease

As discussed in the previous section, the hallmark features of AD are the accumulation of extracellular Aβ and intracellular hyperphosphorylated tau [6,23,24,31,32,43,118]. Beyond mere accumulation, Aβ undergoes various biochemical PTMs that alter its structure and function, thereby exacerbating its toxicity [18,31,94]. Similarly, tau is subject to multiple PTMs in addition to hyperphosphorylation, which can further influence its functional properties and contribute to the progression of AD pathology [18,31,43,59,94]. These observations suggest that PTMs represent a critical mechanistic component in the pathogenesis of AD [6,23,24,31,32,43,118]. Both modified Aβ and tau are typically cleared by PQC systems [93,111]. However, PTMs can impair PQC efficiency, leading to their accumulation and enhanced toxicity. Consequently, while Aβ-lowering therapies may reduce the overall substrate available for PTMs, the remaining Aβ can still undergo modification and exert deleterious effects, albeit at reduced levels [20,112,113]. Therefore, Aβ-lowering strategies may slow disease progression but are unlikely to completely halt it, and PTMs and PQCs should be considered to get a better therapeutic outcome [18,20,31,79,80,93,94,110,112,113,126]. In this section, we discuss the roles of PTMs in modulating Aβ and tau function in the context of AD pathology, as well as the impact of PQC systems on these pathological processes.

### 6.1. Posttranslational Protein Modification on the Pathology of AD

**Figure 1:**
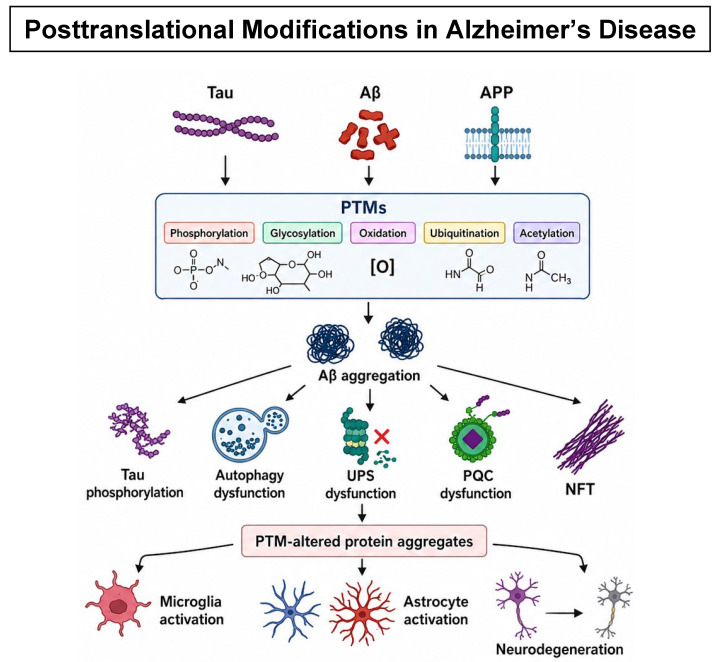
Potential role of posttranslational modifications in AD pathology. Aβ deposition has long been considered a central driver of AD pathology. However, its presence in cognitively normal individuals indicates that Aβ accumulation alone is insufficient to initiate disease progression. Hence, qualitative changes such as PTMs could be critical in triggering and propagating the pathology. PTMs influence multiple aspects of APP processing and Aβ biology, with glycosylation regulating APP trafficking and proteolytic cleavage, thereby modulating Aβ generation. Once formed, Aβ undergoes several PTMs, including phosphorylation, oxidation, and ubiquitination. Such changes alter its structural conformation, and enhance aggregation propensity, as well as increase resistance to degradation. These modified Aβ species might be poorly handled by PQC systems, including molecular chaperones, UPS, and autophagy–lysosomal systems, leading to progressive impairment of these systems and the accumulation of misfolded protein aggregates. The resulting aggregates disrupt cellular homeostasis, activate microglia and astrocytes, and promote chronic neuroinflammation. They can also exert direct neurotoxic effects that contribute to synaptic dysfunction and neuronal loss. In parallel, tau protein undergoes extensive PTMs, particularly hyperphosphorylation, which reduces its affinity for microtubules, promotes aggregation, and leads to the formation of NFTs. Notably, Tau phosphorylation impairs its degradation. Together, these processes highlight that PTMs are key modulators of both Aβ and tau pathology, driving a self-perpetuating cycle of proteostatic failure, neuroinflammation, and neurodegeneration that underlies the progression of AD. APP = amyloid precursor protein; Aβ = amyloid β peptide; PTMs = posttranslational modifications; UPS = ubiquitin–proteasome system; PQC = protein quality control systems; NFT = neurofibrillary tangles.

Several post-translational modifications in Aβ and tau, including phosphorylation, acetylation, ubiquitination, methylation, and oxidation, have been reported (Figure 1), with many of them being associated with specific pathological conditions [18,31,43,76,79,80,87,94,95,96,97,108]. The enzymes related to posttranslational modifications and their functions that are implicated in AD are listed in Table 1. The details of these post-translational modifications are discussed below.

(a) Phosphorylation: Among the posttranslational modifications, hyperphosphorylation of tau proteins has been extensively investigated, and its role in AD is well understood [55,56,57]. Enzymes like glycogen synthase kinase 3β (GSK-3β), mitogen-activated protein kinases, and cyclin-dependent kinase 5 (CDK5) are involved in tau phosphorylation, while phosphatases like protein phosphatase 2A (PP2A) are responsible for dephosphorylation [58,59,60]. Dysregulation of these enzymes contributes to tau pathology and is suggested to be important in AD [61,62,63].

In addition to tau phosphorylation, studies have shown that phosphorylation also plays a critical role in Aβ-mediated pathology [43,58,59,81,127]. For instance, phosphorylation of APP at specific sites influences its pathophysiological functions [81]. Key phosphorylated residues in APP include Tyr653, Ser655, Thr668, Ser675, Tyr682, Thr686, and Tyr687, with Thr668 being the most extensively studied [43,58,81]. The phosphorylation of APP at Thr668 occurs predominantly in the brain, and its regulation, as well as its functional implications, have been well-documented [43,58,59]. Several kinases, such as neuronal cyclin-dependent kinase 5 (cdk5), p34cdc2 protein kinase (cdc2), glycogen synthase kinase-3β (GSK-3β), and c-Jun N-terminal kinases, are implicated in APP phosphorylation. Additionally, Fyn tyrosine kinase has been identified as responsible for phosphorylating Tyr682 [43,58,81].

Functionally, APP phosphorylation acts as a molecular switch that shifts its processing from the non-amyloidogenic to the amyloidogenic pathway, thereby accelerating Aβ production and plaque formation [81,127,128]. Phosphorylated APP exhibits altered intracellular trafficking, such as accumulation in early endosomes rather than progressing through the Golgi network. In AD brains, phosphorylation at Thr668 is markedly elevated and enhances APP interaction with BACE1 within early endosomes [81,127,128]. Such interactions promote amyloidogenic cleavage and increase the generation of neurotoxic Aβ species. Phosphorylation at Ser675 further contributes to pathology by suppressing α-secretase-mediated non-amyloidogenic processing. Emerging evidence also indicates that phosphorylated APP facilitates tau pathology by promoting tau uptake, propagation, and subsequent hyperphosphorylation. Hence, APP phosphorylation could contribute to neurofibrillary tangle formation [43,58,59,61,81,83,99,127,128,129].

In addition to APP, Aβ itself undergoes phosphorylation at residues such as serine-8, tyrosine-10, and serine-26 [127,129]. Protein kinase A is shown to have the ability to phosphorylate Aβ. Phosphorylated Aβ has been detected in AD brains, indicating its significant role in disease pathology [43,58,81]. Phosphorylation at serine-8 and serine-26 significantly alters the biophysical and toxic properties of Aβ by enhancing β-sheet formation, promoting aggregation, and stabilizing oligomeric and fibrillar assemblies [129]. For example, serine-26 phosphorylation is associated with the formation of highly stable, aggregation-prone species that are resistant to proteolytic degradation, while serine-8 phosphorylation increases seeding capacity and accelerates plaque formation [43,59,81,127,128,129]. These phosphorylated Aβ species exhibit enhanced synaptotoxicity, disrupt neuronal signaling, and contribute to oxidative stress and neuroinflammation. Moreover, their clearance by PQC systems is often impaired, facilitating their accumulation in the brain [43,59,81,93,112,113,126,127,128,129].

Importantly, phosphorylation-dependent modifications at both the APP and Aβ levels may act as a feed-forward pathogenic loop that enhances Aβ production, aggregation, and persistence, while also promoting downstream tau pathology [24,32,43,118,128]. These findings highlight phosphorylation as a central post-translational mechanism linking amyloid and tau pathologies in AD. Notably, such modification-specific processes are not directly targeted by current Aβ-lowering strategies, which primarily reduce total Aβ burden without addressing its pathogenic biochemical states. Therefore, therapeutic approaches aimed at modulating phosphorylation events or selectively targeting modified Aβ species may be required to more effectively attenuate disease progression in the brain [43,59,81,93,112,113,126,127,128,129].

(b) Ubiquitination: Ubiquitination is another protein modification implicated in AD, affecting both Aβ and tau proteins [91]. In AD, ubiquitinated tau and Aβ peptides accumulate, indicating proteasomal dysfunction [89,91]. In the context of Aβ, E3 ligases such as the C-terminus of Hsc70-interacting protein (CHIP) and Parkin are particularly important [92]. These E3 ligases recognize misfolded or aggregated Aβ peptides [93]. For instance, CHIP specifically binds to and ubiquitinates toxic Aβ species [45]. In normal conditions, soluble tau is degraded by the proteasome [89,91]. E3 ubiquitin ligases, including CHIP, are vital in targeting misfolded or hyperphosphorylated tau [92]. Parkin has also been shown to contribute to tau ubiquitination [93]. Importantly, a nonhereditary mutant protein, UBB + 1, is consistently found in postmortem AD brains, especially in sporadic cases [91]. Although UBB + 1 can be conjugated to target proteins, it impairs their proteasomal degradation [89,91]. The accumulation of UBB + 1 conjugated with tau is a hallmark of AD and other tauopathies, such as frontotemporal dementia and progressive supranuclear palsy [92].

Mechanistically, impaired ubiquitination and proteasomal dysfunction potentially contribute to AD pathology through several interconnected pathways [86,88,89,90,91,92,93,105]. First, defective clearance of ubiquitinated Aβ and tau, especially with BBB + 1, leads to their intracellular accumulation, promoting aggregation and toxicity. Second, proteasome impairment results in the accumulation of misfolded and damaged proteins, exacerbating proteotoxic stress and overwhelming cellular quality control systems. Third, dysregulated ubiquitination may alter the balance between protein degradation and aggregation, favoring the formation of insoluble, seeding-competent species [86,88,89,90,91,92,93,105]. In addition, ubiquitin-dependent signaling pathways involved in synaptic function, mitochondrial homeostasis, and neuroinflammation may be disrupted, further contributing to neuronal dysfunction. Collectively, these mechanisms highlight the critical role of ubiquitination in maintaining proteostasis, and its disruption as a central driver of Aβ and tau accumulation, neurotoxicity, and disease progression in AD [86,88,89,90,91,92,93,105].

(c) Acetylation: Protein acetylation is a fundamental regulator of neuronal plasticity, memory, and learning [96]. In the context of AD, the disruption of acetyl-homeostasis, manifesting as both histone hypoacetylation and non-histone hyperacetylation, impairs physiological homeostasis and facilitates the accumulation of proteopathic proteins [98]. Recent research underscores protein acetylation as a critical driver of AD pathology, influencing everything from gene transcription to the stability of the synaptic proteome [82]. As the primary epigenetic regulator of gene expression, histone acetylation is frequently compromised in the AD brain [79,94]. Evidence suggests that HATs and HDACs enzymes undergo significant, region-specific alterations: 1. Frontal Cortex: Studies indicate a significant depletion of HATs, including CREB-binding protein (CBP) and p300/CBP-associated protein (PCAF), alongside reductions in HDAC1 and HDAC2 (notably, HDAC3 levels remain stable) [98]. 2. Hippocampus: HDAC1 levels are significantly decreased, while CBP exhibits a consistent downward trend [79,94]. 3. Parietal Cortex: Levels of the Class III deacetylase SIRT1 are reduced in human AD subjects, though it is noted that this depletion is not always replicated in murine AD models [96].

While HDAC inhibitors have demonstrated neuroprotective potential by rescuing memory and synaptic function in AD models, the landscape is complex; for instance, the HAT p300 can upregulate PS1 expression, subsequently increasing Aβ production [98]. Beyond the epigenome, the acetylation of non-histone proteins, most notably tau, is a decisive event in AD progression [82]. Acetylation of tau at specific lysine residues occurs early in the neurodegenerative process and fundamentally alters its clearance [79,94]. While tau is typically degraded via the proteasome and chaperone-mediated autophagy (CMA), acetylation reroutes the protein toward macro-autophagy and endosomal micro-autophagy [98]. This shift disrupts tau homeostasis in an isoform-specific manner: For example, in 4R Tau, acetylation generally inhibits aggregation, while 3R Tau acetylation, specifically at Lysine 298, promotes aggregation [82]. Abnormal acetylation at K274 and K281 is directly linked to the synaptic failure observed in AD [79,94]. This modification leads to a reduction in KIBRA [98]. KIBRA serves as a bridge between the actin cytoskeleton and synaptic signaling by regulating cytoskeletal dynamics. It interacts with Dendrin and Synaptopodin to maintain dendritic spine morphology. It also stabilizes synaptic AMPA receptors through facilitating the trafficking and stabilization of GluA1/GluA2 subunits. The depletion of KIBRA, driven by aberrant tau acetylation, disrupts the stabilization of AMPA receptors, thereby impairing fast excitatory synaptic transmission and the maintenance of synaptic strength [82].

(d) Glycosylation: Protein glycosylation plays a significant role in AD pathology, affecting both APP and tau [103,104]. Glycosylation of APP, particularly O-glycosylation, is critical for its processing into Aβ [87,130]. Alterations in glycosylation patterns influence APP processing, with highly O-glycosylated APP being more prone to cleavage, leading to Aβ production [102,108]. Additionally, the glycosylation of Aβ-producing enzymes, like BACE1, affects their activity and enhances Aβ production [101,102].

In the case of tau, glycosylation contributes to its hyperphosphorylation and aggregation into paired helical filaments [77,87,102]. Aberrant tau glycosylation has been detected in AD patients, even at early stages, particularly N-glycosylation, which is not observed in healthy brains [77,87,102]. Importantly, N-glycosylation appears to trigger early tau hyperphosphorylation in AD, as confirmed by in vitro studies [104]. Tau mutants lacking N-glycosylation sites (N167Q, N359Q, and N410Q) show site-dependent regulation of phosphorylation, highlighting the role of early N-glycosylation in driving tau pathology [95,104]. Glycosylated tau is more susceptible to phosphorylation by cAMP-dependent protein kinase, suggesting a link between glycosylation and tau hyperphosphorylation, a key feature in neurofibrillary tangle formation. O-glycosylation of tau is also prevalent in human brains, and a decrease in O-GlcNAc glycosylation has been noted in AD, which negatively correlates with tau phosphorylation [95,97,99,100]. Overall, changes in glycosylation patterns at different stages of tau modification may contribute to AD progression, with aberrant glycosylation playing a key role in triggering tau hyperphosphorylation and neurofibrillary tangle formation [77,87,102].

(e) Oxidation: Numerous studies have demonstrated that oxidative stress and protein oxidation are common features of AD [76,85,109]. One study found that methionine 35 of Aβ can be oxidized by hydrogen peroxide, which reduces its conversion into fibrils [72]. However, the study did not assess the levels of toxic soluble oligomers, so it remains unclear whether Aβ oxidation by hydrogen peroxide actually reduces Aβ-induced toxicity [73]. Transition metals like copper and iron, which are found in amyloid plaques, are known to bind Aβ and generate reactive oxygen species (ROS) [74]. This metal-induced ROS production has been linked to the self-oxidation of Aβ amino acids, such as histidine and phenylalanine [75]. Chelators like deferoxamine, which bind these metals, have been shown to slow AD progression [104]. Additionally, peroxidase oxidation causes the formation of stable Aβ dimers through di-tyrosine bridges, which are neurotoxic [104,130]. These findings suggest that oxidative modifications of Aβ play a crucial role in AD pathology [76,85,109]. Therefore, therapeutic strategies should target not only Aβ production and aggregation but also its oxidative modifications [72].

Tau protein also undergoes oxidation, which is a key pathological modification in AD [76,85,109]. Tau oxidation, primarily at cysteine residues, alters its conformation, stability, and function [72]. The redox state is particularly important in the process of tau fibril formation [73]. For instance, cysteine oxidation has been shown to facilitate disulfide bond formation in four-repeat tau, leading to the creation of structurally compact monomers [74]. These monomers can form fibrils, though they are less stable than those formed under reducing conditions [75]. Importantly, these fibrils can break into seeds, which promote the formation of stable tau fibrils [76,85,109]. Thus, oxidative stress may act as a trigger for tau fibrillary aggregation, contributing to AD progression [72].

### 6.2. PQC on the Pathology of AD

**Figure 2:**
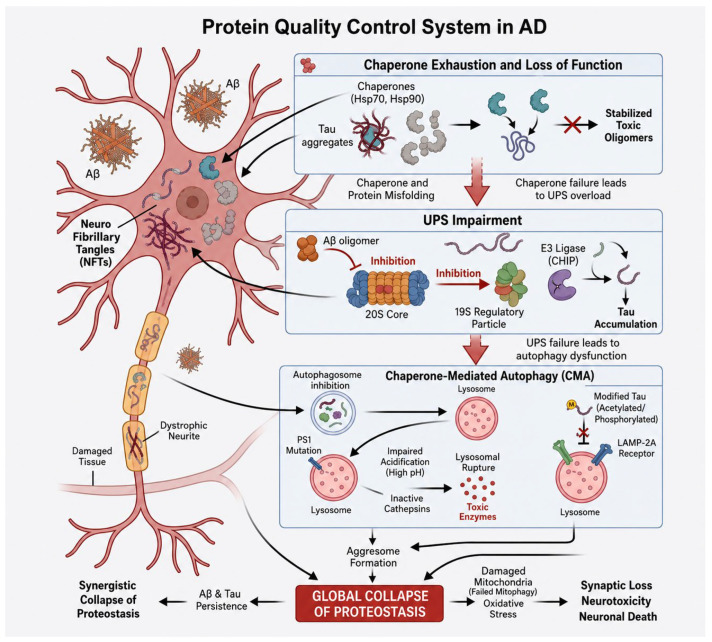
Potential role of protein quality control systems in AD pathology. In the brains of individuals with AD, multiple PQC systems are significantly compromised, contributing to the accumulation of misfolded and aggregated proteins. It has been shown that posttranslationally modified forms of Tau and Aβ promote the sequestration of key molecular chaperones, including Hsp70 and Hsp90, into tau aggregates and Aβ oligomers. This sequestration results in a functional depletion of available chaperones, thereby impairing the proper folding of newly synthesized and stress-denatured proteins. In addition, Aβ oligomers have been shown to directly interact with and inhibit the UPS, further reducing the capacity of the cells to degrade abnormal proteins. Given that posttranslational modifications influence Aβ aggregation and oligomerization, these modifications may critically determine the extent of proteasomal impairment. Additionally, dysfunction of the autophagy–lysosomal pathway is a well-established feature of AD. Such dysfunction could exacerbate the accumulation of toxic protein species. Collectively, these impairments lead to a collapse of cellular proteostasis, a tightly regulated network essential for maintaining protein homeostasis. Disruption of proteostasis is closely linked to the stress responses and activation of neuroinflammatory pathways and the progression of neurodegeneration, suggesting that PQC failure establishes a self-amplifying cycle that drives the pathogenesis and progression of AD. PQC = protein quality control system; UPS = ubiquitin–proteasome system; Hsp = heat-shock protein.

In AD, the accumulation of Aβ plaques and NFTs is fundamentally a failure of the PQC system [20,93,110,112,130]. The chronic imbalance between the production of these proteopathic species and their clearance leads to a collapse of cellular proteostasis, triggering a cascade of neurotoxicity, synaptic loss, and eventual neuronal death (Figure 2). The enzymes related to PQC, and their functions that are implicated in AD, are listed in Table 2.

#### 6.2.1. Chaperone Exhaustion and Loss of Function

While molecular chaperones are initially upregulated to counteract protein misfolding, the chronic stress on the system in AD eventually exhausts the chaperone network. [110,118,131]. Key chaperones, including Hsp70 and Hsp90, are frequently found sequestered into tau aggregates and Aβ oligomers [115,119]. This sequestration reduces the pool of available chaperones to assist the de novo folding of other essential proteins [132]. Consequently, the Hsp70 co-chaperone system, which normally maintains tau solubility, becomes overwhelmed [120,121,133]. Importantly, Hsp70 exerts a pivotal anti-inflammatory and neuroprotective role by modulating stress-responsive signaling pathways, including suppression of NF-κB activation and attenuation of pro-inflammatory cytokine production in glial cells [115,134]. Through these mechanisms, Hsp70 limits chronic neuroinflammation and protects neurons from inflammation-induced toxicity [120,121,133,135]. In addition, Hsp70 can inhibit apoptosis and stabilize mitochondrial function, thereby mitigating neurodegenerative processes. However, in AD, functional exhaustion of Hsp70 compromises these protective effects, contributing to sustained neuroinflammation and neuronal vulnerability [115,119]. Furthermore, Hsp70 and Hsp90, when bound to tau and Aβ, interact with co-chaperones such as BAG-1 and CHIP that link client proteins to the proteasome, thereby facilitating their degradation [115,120,121,133,135]. When chaperones fail to refold tau or direct it for degradation, they may instead stabilize toxic oligomeric intermediates that act as seeds for further aggregation [19,110,115,116,117,120,121,131,136].

#### 6.2.2. UPS Impairment

The UPS is significantly compromised in AD, creating a vicious cycle of protein accumulation [89,91]. Research indicates that Aβ oligomers can directly bind to and inhibit the catalytic 20S core of the proteasome [91,92,93]. Similarly, highly branched polyubiquitin chains on tau can inhibit the 19S regulatory particle, preventing the translocation and degradation of other substrates [114,116]. Moreover, mutations or oxidative damage to E3 ligases, such as CHIP, impair the tagging of misfolded tau, allowing it to evade proteasomal recognition and accumulate in the cytoplasm [115,121]. Impaired UPS activity not only promotes the buildup of toxic Aβ and tau species but also can disrupt the turnover of key regulatory proteins involved in synaptic function, cell cycle control, and stress responses, thereby contributing to synaptic dysfunction and neuronal loss. Furthermore, UPS failure can trigger compensatory activation of autophagy; however, this pathway is often insufficient or itself compromised in AD, further amplifying protein accumulation. The progressive decline in proteasomal function thus reinforces a self-perpetuating cycle in which accumulating aggregates increasingly inhibit UPS activity, accelerating disease progression and neurodegeneration [20,86,88,89,90,91,105,106,107,122].

#### 6.2.3. Defective Autophagy–Lysosomal Flux

The autophagy–lysosome pathway (ALP) is often considered the backup for the UPS when dealing with large aggregates [105]. However, in AD, the ALP is stalled at multiple stages: 1. Autophagosome Accumulation: AD brains show a massive accumulation of immature autophagosomes, particularly in dystrophic neurites. This suggests a failure in the fusion of autophagosomes with lysosomes, likely due to disrupted axonal transport. 2. Lysosomal Acidification: Mutations in Presenilin-1 (PS1) disrupt the acidification of the lysosomal lumen. Without an acidic environment, lysosomal proteases (cathepsins) remain inactive, leading to the buildup of undigested cargo and the eventual rupture of the lysosome, which releases toxic enzymes into the cytoplasm [100]. 3. CMA Failure: Chaperone-mediated autophagy (CMA) is also hindered; while tau contains the KFERQ-like motif for CMA recognition, its post-translational modifications (such as acetylation and phosphorylation) can prevent its translocation through the LAMP-2A receptor, rerouting it toward more error-prone degradation pathways [106,107,122,123,124,125]. These defects collectively impair the clearance of aggregation-prone proteins, leading to the progressive accumulation of toxic Aβ and tau species [31,32,44]. In addition, defective ALP contributes to synaptic dysfunction by disrupting organelle turnover and impairing neuronal homeostasis, while lysosomal leakage can activate inflammatory and cell death pathways. The failure of autophagic flux also exacerbates proteotoxic stress and oxidative damage, further promoting neurodegeneration. Thus, ALP dysfunction not only compromises aggregate clearance but also amplifies multiple pathogenic cascades, reinforcing disease progression in AD [106,107,122,123,124,125].

#### 6.2.4. Synergistic Collapse of Proteostasis

The failure of these three systems is not isolated [89,91]. UPS impairment forces a heavier load onto the ALP, which, if already compromised by lysosomal dysfunction, leads to the formation of aggresomes [20,86,88,89,90,91,105,106,107,122]. This global collapse of PQC not only allows Aβ and tau to persist but also prevents the degradation of damaged mitochondria (mitophagy), further increasing oxidative stress and fueling the neurodegenerative cycle [100,101,102,103,104,105,106,107,108,109,110,111,112,113,114,115,116,117,118,119,120,121].

### 6.3. Bidirectional Effects of PTMs and PQCs on AD

PTMs and PQC systems interact bidirectionally to regulate protein fate [18,20,52,79,80,93,94,111,112,113,126]. PTMs such as phosphorylation, acetylation, oxidation, glycosylation, and ubiquitination alter the conformation, stability, and aggregation propensity of Aβ and tau, thereby influencing their recognition and clearance by PQC pathways. For example, phosphorylation and oxidation promote aggregation and reduce degradability, while acetylation can inhibit ubiquitination and redirect proteins away from proteasomal or chaperone-mediated degradation [4,5,10,11,13,43,94]. Conversely, PQC systems—including chaperones, the ubiquitin–proteasome system (UPS), and autophagy—control the turnover of PTM-modified proteins and help limit their accumulation. Notably, aggregated Aβ can directly inhibit proteasomal activity, further impairing UPS function. When PQC capacity is compromised, modified proteins accumulate and undergo further PTMs, enhancing their toxicity approaches [18,20,31,77,79,80,87,93,94,98,108,111,112,113,117]. Thus, PTMs can impair PQC efficiency, while PQC dysfunction promotes the persistence and propagation of aberrantly modified proteins, forming a self-reinforcing cycle.

## 7. Therapeutic Implications of PQC and PTMs in AD

**Figure 3:**
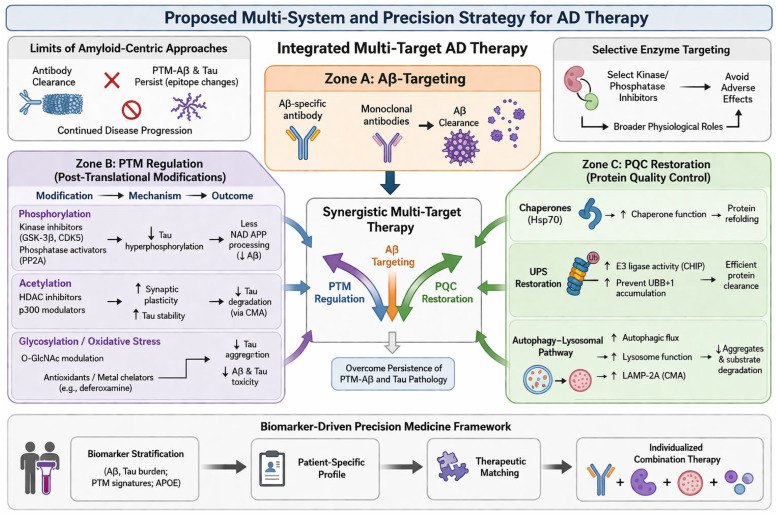
A Precision Multi-Target Strategy for AD Therapy. Antibody-mediated clearance of Aβ currently represents the most advanced therapeutic approach for AD. However, these strategies have shown limited efficacy in halting disease progression. One plausible explanation is that PTMs of Aβ and tau can mask antibody-recognized epitopes, thereby reducing the efficiency of clearance mechanisms and allowing pathogenic species to persist. To address this limitation, therapeutic strategies should be expanded to include interventions targeting PTMs. For instance, antioxidants such as astaxanthin or vitamin E may help mitigate oxidative stress-induced protein modifications, thereby preserving protein integrity and enhancing therapeutic responsiveness. Similarly, kinase inhibitors may be employed to reduce aberrant phosphorylation. However, given the broad physiological roles of kinases, the use of highly selective inhibitors is essential to minimize off-target effects. In parallel, disruptions in protein quality control (PQC) systems, including the ubiquitin–proteasome system and autophagy–lysosomal pathways, also contribute to the accumulation of misfolded and modified proteins in AD. Therefore, restoring PQC function represents another critical therapeutic axis. Importantly, the extent and type of PTMs and PQC dysregulation vary among patients. This highlights the need for biomarker-driven stratification to identify patient-specific molecular alterations. Tailoring therapeutic interventions based on individual PTM and PQC profiles may maximize treatment efficacy, reduce adverse effects, and ultimately improve clinical outcomes and quality of life for patients with AD. ↑ = increased; ↓ = decreased.

The complex interplay between PTMs and PQC systems in AD reveals multiple therapeutic opportunities beyond conventional amyloid-centric approaches [18,20,31,77,79,80,87,93,94,98,108,111,112,113,117]. Although antibody-mediated Aβ clearance has shown clinical promise, PTM-modified Aβ species and tau may persist due to epitope alterations, allowing these variants to continue driving disease progression. Consequently, effective treatment requires integrated strategies targeting both PTM dysregulation and PQC dysfunction [18,20,31,79,80,93,94,108,111,112,113,117]. A comprehensive strategy to address both PTM and PQC dysregulations is shown in Figure 3.

Modulation of phosphorylation represents a key therapeutic avenue, given its central role in both Aβ and tau pathology. Inhibition of kinases such as GSK-3β and CDK5, or activation of phosphatases like PP2A, may reduce tau hyperphosphorylation and aggregation [31,59,61,87]. Similarly, targeting APP phosphorylation may shift its processing toward the non-amyloidogenic pathway, thereby reducing Aβ production. However, given the broad physiological roles of these enzymes, selective targeting is essential to minimize adverse effects.

Targeting acetylation has also shown therapeutic promise. Isoform-selective inhibition of histone deacetylases (HDACs), as well as modulation of acetyltransferases such as p300, may restore synaptic plasticity and regulate tau stability [82,87,96,98,99,100,101]. In addition, preventing pathological tau acetylation or restoring its degradation via chaperone-mediated autophagy (CMA) may directly mitigate tau toxicity. Interventions targeting glycosylation represent another emerging strategy; modulation of O-GlcNAcylation can reduce tau aggregation, while regulation of APP and BACE1 glycosylation may alter amyloidogenic processing [82,87,96,98,99,100,101].

Because oxidative stress drives many pathological PTMs, antioxidant therapies and metal chelators, such as deferoxamine, may limit Aβ and tau aggregation and toxicity by attenuating redox-mediated damage [20,23,75,76,85,108,109,111]. Concurrently, restoration of PQC systems is critical for re-establishing proteostasis. This includes pharmacological induction of molecular chaperones (e.g., Hsp70) to enhance protein refolding and activation of proteasomal function to alleviate protein accumulation [115]. Given the frequent impairment of the ubiquitin–proteasome system (UPS) in AD, restoring ubiquitination through E3 ligases such as CHIP, or preventing the accumulation of aberrant ubiquitin species like UBB + 1, is essential for efficient clearance of misfolded proteins [20,86,88,89,90,91,92]. Augmentation of the autophagy–lysosomal pathway, through stimulation of autophagic flux, promotion of autophagosome–lysosome fusion, and upregulation of LAMP-2A, may further enhance degradation of aggregated substrates [106,107,122].

While monoclonal antibodies targeting Aβ remain a central therapeutic approach, their modest efficacy may reflect the persistence of PTM-modified Aβ species and downstream tau pathology. Therefore, combination therapies integrating immunotherapy with PTM-targeting and PQC-restoring strategies are likely to provide greater clinical benefit [4,5,10,11,13,43,94]. Importantly, the selection of therapeutic targets should be guided by patient-specific disease profiles. Stratification based on biomarkers—such as Aβ and tau burden, PTM signatures, and genetic factors, including APOE genotype—may enable more precise therapeutic matching. Such individualized, biomarker-driven approaches, integrating multi-target interventions, represent a promising path toward achieving sustained disease modification in AD.

## 8. Current Knowledge Gaps and Future Perspectives: The Interplay of PTMs and PQC in AD

Despite significant advancements in mapping the PTM landscape and the failure of the PQC system in AD, several critical questions remain unanswered. Addressing these gaps is essential for moving beyond descriptive pathology toward the development of precise, disease-modifying interventions [18,20,31,77,79,80,87,93,94,98,108,111,112,113,117].

### 8.1. The PTM Crosstalk and Temporal Hierarchy

A primary unknown is the hierarchical crosstalk between different PTMs [6,44,76,94]. While it is established that phosphorylation, acetylation, and glycosylation all occur on tau and APP, the temporal sequence of these modifications remains elusive [18,20,31,77,79,80,87,93,94,98,108,111,112,113,117]. It is unclear which PTM serves as the primary trigger that initiates the cascade of misfolding, and which are secondary consequences of cellular stress [6,44,76,94]. For example, does O-glycosylation loss always precede hyperphosphorylation, or is their relationship stochastic [95,97,99,100]? Understanding this sequence is vital for identifying the earliest possible therapeutic windows. Furthermore, we lack a complete understanding of how one PTM might sterically hinder or promote another on the same protein backbone, a phenomenon known as the PTM code.

### 8.2. Subcellular PQC Heterogeneity and Selective Vulnerability

While we understand the global failure of the UPS and ALP, the reasons behind selective neuronal vulnerability remain poorly understood [118,119,132,133]. It is currently unknown why specific neuronal populations, such as those in the entorhinal cortex, succumb to PQC failure and PTM-driven aggregation significantly earlier than others [118,119,120,121,122]. We have yet to determine if this is due to cell-type-specific differences in chaperone stoichiometry or localized variations in the lysosomal pH environment [99,100,101]. Additionally, the role of PQC within specific subcellular compartments, such as the distal axon versus the soma, requires further investigation to explain why protein clogging often initiates in the periphery of the neuron [20,86,88,89,90,91,105,106,107,122].

### 8.3. Transition from Soluble to Toxic Species

A significant gap exists in our understanding of how PQC enzymes distinguish between benign soluble proteins and the toxic oligomers that are now considered the primary drivers of neurotoxicity [20,89,91,93,111,112,113]. While E3 ligases like CHIP and Parkin are known to target misfolded proteins, the specific structural motifs they recognize in transient, non-fibrillar Aβ or tau intermediates remain unidentified [110,114,115,120,132]. It is also unknown whether PTMs like oxidation or nitration create neo-epitopes that allow these toxic species to actively evade PQC detection or if they simply overwhelm the system through sheer volume.

### 8.4. Translating Bench to Bedside: The Specificity Challenge

Finally, a major hurdle in AD research is the lack of PQC-targeted therapies that do not disrupt healthy cellular functions. Current HDAC inhibitors and autophagy enhancers often lack the specificity required to target only the pathological isoforms of tau or Aβ [106,107,122]. Determining how to selectively upregulate the degradation of modified proteins while leaving the native protein pool intact remains the holy grail of PQC-based drug development [106,107]. Future research must focus on the structural biology of PTM-specific PQC receptors to achieve this necessary precision.

## 9. Conclusions

AD develops through the combined effects of abnormal protein modifications and failure of the protein quality control system [18,31,80,93,94,98,112,113]. PTMs directly change the behavior of Aβ and tau, increasing their aggregation and toxicity. At the same time, PQC pathways fail to remove these harmful proteins. This failure leads to protein accumulation, neuroinflammation, and neuronal death. The interaction between PTMs and PQC dysfunction creates a self-reinforcing cycle that drives disease progression [18,31,80,93,94,98,112,113]. Therefore, both systems should be considered together when developing new therapies. Future studies should focus on identifying the order of PTMs, understanding why certain neurons are more vulnerable, and designing targeted treatments. Approaches that restore protein balance without affecting normal cellular functions may offer more effective and precise treatments for AD.

## Figures and Tables

**Table 1:** Posttranslational modifications in AD pathology.

PTM	Key Enzymes/Factors	Major Consequences
**Phosphorylation**	GSK-3β, CDK5, MAPKs, JNK, Fyn; PP2A	Tau hyperphosphorylation → NFTs; altered APP processing (↑ Aβ); modified Aβ aggregation and toxicity
**Ubiquitination**	CHIP, Parkin, UBB + 1	Impaired proteasomal degradation; accumulation of ubiquitinated tau/Aβ; UBB + 1 inhibits UPS → promotes tau deposition and proteotoxic stress
**Acetylation**	HATs (CBP, p300, PCAF); HDACs (HDAC1/2/3, SIRT1)	Dysregulated gene expression; altered tau clearance; synaptic dysfunction (↓ KIBRA, impaired AMPAR stability)
**Glycosylation**	Glycosyltransferases; BACE1 (modified)	Enhanced APP cleavage (↑ Aβ); ↓ O-GlcNAc promotes tau hyperphosphorylation and aggregation
**Oxidation**	ROS, H_2_O_2_, Cu/Fe, peroxidases	Aβ oxidative modification (toxic dimers); tau oxidation → aggregation and fibril seeding

↑ indicates increased, and ↓ indicates decreased.

**Table 2:** Protein quality control systems in AD pathology.

PTM/Modification Type	Enzymes/Factors Involved	Major Consequences
**Ubiquitination**	E3 ligase CHIP; proteasome (20S core, 19S regulatory particle)	Impaired ubiquitin tagging and proteasomal degradation; accumulation of misfolded tau; inhibition of proteasome by Aβ and polyubiquitinated tau
**Phosphorylation/Acetylation (tau PTMs affecting CMA)**	Kinases, acetyltransferases (not specified); LAMP-2A receptor (CMA)	PTMs block tau recognition/translocation via CMA; rerouting to inefficient degradation pathways; enhanced tau accumulation
**Protein misfolding (chaperone-associated modification state)**	Hsp70, Hsp90, co-chaperones	Chaperone sequestration into aggregates; reduced folding capacity; stabilization of toxic oligomeric intermediates
**Proteolytic processing (lysosomal**	Cathepsins (lysosomal proteases); PS1 (lysosomal acidification regulator)	Impaired lysosomal degradation due to defective acidification; accumulation of autophagosomes and undigested cargo; lysosomal rupture and cytotoxicity
**degradation)**		
**Oxidative modification (indirect PTM)**	Reactive oxygen species (ROS); damaged E3 ligases (e.g., CHIP)	Oxidative damage to PQC components; impaired ubiquitination; increased proteotoxic stress and aggregation
**Polyubiquitin chain modification (aberrant ubiquitin signaling)**	Polyubiquitin chains; proteasome 19S subunit	Inhibition of substrate translocation into proteasome; global UPS dysfunction; accumulation of aggregation-prone proteins

## Data Availability

No new data were created or analyzed in this study. Data sharing is not applicable to this article.
